# Epilepsia Open—June 2022—Announcements

**DOI:** 10.1002/epi4.12609

**Published:** 2022-06-01

## ILAE Congresses:

XVI Workshop on Neurobiology of Epilepsy (WONOEP 2022)

4–8 July 2022

Talloires, France

www.ilae.org/wonoep2022

14th European Epilepsy Congress

9–13 July 2022

Geneva, Switzerland

www.ilae.org/eec2022

5th Dianalund Summer School on EEG and Epilepsy

16–23 July 2022

Dianalund, Denmark

https://www.ilae.org/congresses/5th‐dianalund‐summer‐school‐on‐eeg‐and‐epilepsy

11th Summer School for Neuropathology and Epilepsy Surgery (INES 2022).

8–11 September 2022

Erlangen, Germany

https://www.ilae.org/congresses/11th‐international‐summer‐school‐for‐neuropathology‐and‐epilepsy‐surgery‐ines‐2021

XII Congreso Latinoamericano de Epilepsia (LAEC 2022)

1–4 October 2022

Colombia

www.ilae.org/laec2022

14th Asian & Oceanian Epilepsy Congress

17–19 November 2022

Virtual congress

www.ilae.org/aoec2022

35th International Epilepsy Congress

2–6 September 2023

Dublin, Ireland

www.ilae.org/iec2023

## Other Congresses:

10th Migrating Course on Epilepsy

17–20 June 2022

Lviv, Ukraine

https://www.ilae.org/congresses/10th‐migrating‐course‐on‐epilepsy

8th Congress of the European Academy of Neurology (EAN)

25–28 June 2022

Vienna, Austria

https://www.ilae.org/congresses/8th‐congress‐of‐the‐european‐academy‐of‐neurology‐ean

Epilepsy Surgery Techniques Meeting (ESTM 2022)

8–9 July 2022

Geneva, Switzerland

https://www.ilae.org/congresses/epilepsy‐surgery‐techniques‐meeting

2022 Advanced San Servolo Epilepsy Course. Bridging Basic with Clinical Epileptology ‐ 7: Accelerating Translation in Epilepsy Research

18–29 July 2022

San Servolo (Venice), Italy

https://www.ilae.org/congresses/2022‐advanced‐san‐servolo‐epilepsy‐course

Irish Epilepsy League Annual Meeting

16 September 2022

Dublin, Ireland

https://www.ilae.org/congresses/irish‐epilepsy‐league‐annual‐meeting1

15th Baltic Sea Summer School on Epilepsy

21–29 September

Virtual school

https://www.ilae.org/congresses/15th‐baltic‐sea‐summer‐school‐on‐epilepsy

Canadian League Against Epilepsy 2022 Scientific Meeting

23–25 September 2022

Kelowna, BC, Canada

https://www.ilae.org/congresses/canadian‐league‐against‐epilepsy‐2022‐scientific‐meeting

5th Swiss Federation of Clinical Neuro‐Societies Congress

28–30 September 2022

Basel, Switzerland

https://sfcns2022.congress‐imk.ch/frontend/index.php

28–30 September 2022

Cleveland Clinic Epilepsy Update and Review Course

Ohio, USA & Virtual course

https://www.clevelandclinicmeded.com/live/courses/EpilepsyUpdate22/

4th Bologna EPIPED‐EEG Course: EEG interpretation in pediatric epilepsies

9–13 October 2022

Bologna, Italy

http://www.epiped‐eeg‐course.com/

2022 ILAE British Branch Annual Scientific Meeting

12–14 October 2022

Cardiff, UK

https://www.ilaebritishconference.org.uk/

Epilepsy Society of Australia 36th Annual Scientific Meeting

26–28 October 2022

Adelaide, Australia

https://www.ivvy.com.au/event/ESA2022/

12th World Congress for Neurorehabilitation

14–17 December 2022

Vienna, Austria & Virtual congress

https://www.wfnr‐congress.org/

## 2023

12th EPODES—BASIC

23–26 January 2023

Brno, Czech Republic

https://www.ilae.org/congresses/12th‐epodes‐basic

15th European Pediatric Neurology Society Congress (EPNS): From genome and connectome to cure

20–24 June, 2023

Prague, Czech Republic

https://www.epns.info/epns‐congress‐2023/

